# Effects of microencapsulated plant essential oils on growth performance, immunity, and intestinal health of weaned Tibetan piglets

**DOI:** 10.3389/fvets.2024.1456181

**Published:** 2024-08-20

**Authors:** Xiaolian Chen, Wenjing Song, Pingwen Xiong, Di Cheng, Weiqun Wei, Quanyong Zhou, Chuanhui Xu, Qiongli Song, Huayuan Ji, Yan Hu, Zhiheng Zou

**Affiliations:** ^1^Institute of Animal Husbandry and Veterinary Science, Jiangxi Academy of Agricultural Sciences, Nanchang, China; ^2^Jiangxi Province Key Laboratory of Animal Green and Healthy Breeding, Nanchang, China; ^3^Institute of Animal Science and Fisheries, Gannan Academy of Sciences, Ganzhou, China; ^4^Jiangxi Tianjia Biological Engineering Co., Ltd., Nanchang, China

**Keywords:** Tibetan piglets, microencapsulation, plant essential oils, growth performance, intestinal health

## Abstract

**Introduction:**

Plant essential oils (PEOs) have received significant attention in animal production due to their diverse beneficial properties and hold potential to alleviate weaning stress. However, PEOs effectiveness is often compromised by volatility and degradation. Microencapsulation can enhance the stability and control release rate of essential oils. Whether different microencapsulation techniques affect the effectiveness remain unknown. This study aimed to investigate the effects of PEOs coated by different microencapsulation techniques on growth performance, immunity, and intestinal health of weaned Tibetan piglets.

**Methods:**

A total of 120 Tibetan piglets, aged 30 days, were randomly divided into five groups with four replicates, each containing six piglets. The experimental period lasted for 32 days. The groups were fed different diets: a basal diet without antibiotics (NC), a basal diet supplemented with 10 mg/kg tylosin and 50 mg/kg colistin sulfate (PC), 300 mg/kg solidified PEO particles (SPEO), 300 mg/kg cold spray-coated PEO (CSPEO), or 300 mg/kg hot spray-coated PEO (HSPEO).

**Results:**

The results showed that supplementation with SPEO, CSPEO, or HSPEO led to a notable decrease in diarrhea incidence and feed to gain ratio, as well as duodenum lipopolysaccharide content, while simultaneously increase in average daily gain, interleukin-10 (IL-10) levels and the abundance of ileum Bifidobacterium compared with the NC group (*p* < 0.05). Supplementation with SPEO, CSPEO, or HSPEO significantly elevated serum immunoglobulin G (IgG) levels and concurrently reduced serum lipopolysaccharide and interferon γ levels compared with the NC and PC groups (*p* < 0.05). Serum insulin-like growth factor 1 (IGF-1) levels in the SPEO and HSPEO groups significantly increased compared with the NC group (*p* < 0.05). Additionally, CSPEO and HSPEO significantly reduced jejunum pH value (*p* < 0.05) compared with the NC and PC groups (p<0.05). Additionally, Supplementation with HSPEO significantly elevated levels of serum immunoglobulin M (IgM) and interleukin-4 (IL-4), abundance of ileum Lactobacillus, along with decreased serum interleukin-1 beta (IL-1β) levels compared with both the NC and PC groups.

**Discussion:**

Our findings suggest that different microencapsulation techniques affect the effectiveness. Dietary supplemented with PEOs, especially HSPEO, increased growth performance, improved immune function, and optimized gut microbiota composition of weaned piglets, making it a promising feed additive in piglet production.

## 1 Introduction

The Tibetan pig, also known as the “Zangxiang” pig, is highly renowned for its exceptional meat quality and its remarkable adaptability to the challenging high-altitude environments (1), which has led to its widespread introduction and breeding in various regions (2). The traditional rearing method for Tibetan pigs primarily involves free-range grazing and natural weaning, resulting in relatively low economic efficiency and hindering the development of the Tibetan pig industry. Early weaning of piglets is increasingly recognized and utilized as an effective means to enhance the reproductive performance of sows and accelerate the growth of piglets. However, after weaning, piglets encounter various physiological and environmental changes that can lead to a temporary growth suppression known as “post-weaning syndrome” (3). The early weaning of piglets frequently precipitates complications including diarrheal episodes, compromised growth dynamics, and reduced dietary intake (4). Weaning-induced stress adversely modulates the structural integrity of the intestinal tract in piglets, perturbing the homeostasis of intestinal microbiota and immune responses, thereby compromising the integrity of the mucosal barrier function. This perturbation serves as a foundational physiological mechanism underlying the manifestation of weaning stress syndrome. As the use of antibiotics as growth promoters has been banned in many countries, their primary application in pig farm now focused on preventing and treating post-weaning diarrhea among groups of pigs. This disease represents a significant challenge to the well-being of piglets (5). Given the global initiative to restrict antibiotic use, exploring viable alternatives to antibiotics is crucial. Such alternatives should effectively enhance growth performance, decrease the incidence of diarrhea, and alleviate intestinal barrier damage caused by post-weaning stress. Ultimately, these efforts aim to enhance breeding efficiency and improve the overall health and welfare of Tibetan piglets.

Plant essential oils (PEOs) have received significant attention in animal production due to their diverse beneficial properties. These oils have shown promising effects in improving animal health and performance by exhibiting antimicrobial, anti-inflammatory, and antioxidative activities (6–9). The antimicrobial properties of PEOs help control bacterial and fungal infections in animals. They have also been found to possess antiviral properties, providing effective measures against viral infections. Additionally, these oils contribute to reducing inflammation and oxidative stress, thereby enhancing animal well-being (10). Moreover, PEOs have shown potential in promoting digestion and nutrient absorption in animals (11). Their augmentation of gastrointestinal well-being culminated in amplified feed intake and enhanced feed efficiency (12). Furthermore, these oils contribute to the development of a well-balanced gut microbiota which plays a vital role in overall animal health (13). Despite the numerous benefits of PEOs, there are challenges associated with their application in animals. For example, their high volatility, instability, susceptibility to oxidation, and pungent odor pose significant obstacles (14). These properties make it difficult to effectively preserve the oils, negatively impacting their palatability for animals and resulting in reduced bioavailability.

Microencapsulation technology is a technique that uses natural or synthetic polymers as shell materials to encapsulate solids, liquids, gases, and other substances into tiny droplets or particles (15). The technology of PEOs microencapsulation formulation is widely applied to process PEOs into microcapsules, with the aim of masking the odor or taste of the oils (16), solidifying liquid PEOs (17), and enhancing the resistance of active ingredients. This includes reducing volatile losses, improving heat resistance, light resistance, antioxidant capacity. The goal is to stabilize the active components of the essential oils, allowing them to reach their intended sites of action and achieve targeted release, thereby enhancing bio-availability (18).

The present study was designed to investigate the hypothesis that diverse microencapsulation types could enhance the efficacy of PEOs in improving the growth performance of weaning Tibetan pigs. Parameters such as weight gain, feed conversion ratio, incidence of diarrhea, serum biochemical indices, intestinal pH value, and intestinal microflora were systematically evaluated. This research aimed to elucidate the potential of microencapsulation techniques to optimize the functional attributes of PEOs, thereby promoting the development of in the livestock industry.

## 2 Materials and methods

### 2.1 Raw material preparation

The PEOs, derived from oregano plants and supplied by Jiangxi Tianjia Biological Engineering Co., Ltd. (Nanchang, Jiangxi, China), contains 135 mg/g thymol and 65 mg/g cinnamaldehyde. The PEOs were ejected from a high-speed spray gun, forming tiny droplets. Inside the encapsulation chamber, materials such as silicon dioxide (SiO2), cyclodextrin, and starch are sprayed onto these droplets, causing the PEO to be adsorbed and gain weight, resulting in the solidification of PEO into solidified PEO particles (SPEO). Cold spray-coated PEO (CSPEO) involves melting hydrogenated vegetable oil (e.g., mono-and diglycerides) with PEOs at a temperature of 80°C. By taking advantage of the oil’s solidification upon cooling, the emulsified liquid is sprayed from a heated nozzle into a chamber where it cools and solidifies, creating microcapsules. Hot spray-coated PEO (HSPEO) utilizes substances such as cyclodextrin, starch, and gelatin as coating materials. These coating materials are dissolved in water and mixed with the core material (PEOs). Through dispersion using a high-speed disperser, a water-in-oil emulsion is formed, which is then homogenized using a high-pressure homogenizer. The resulting emulsion is dried in an airflow spray dryer, yielding microcapsules.

### 2.2 Animals, diets, and management

One hundred and twenty newly weaned female Tibetan piglets (30 d, BW 5.14 ± 0.09 kg) provided by Youdao Agricultural Development Co., Ltd., (Ganzhou, Jiangxi, China) were allocated according to initial weight to five dietary treatments, with four replicates (pens) of six animals each. The same basal feed (corn-soybean meal-based) was formulated according to the nutritional requirements for pigs specified in the NRC (2012) guidelines (Table 1). Piglets were fed with a basal diet (NC group), or a basal diet supplemented with 10 mg/kg chlortetracycline and 50 mg/kg colistin sulfate (PC group), a basal diet supplemented with 300 mg/kg SPEO (SPEO group), a basal diet supplemented with 300 mg/kg CSPEO (CSPEO group), or a basal diet incorporated with 300 mg/kg HSPEO (HSPEO group). All the piglets were kept in pens with dimensions of 280 cm long ×160 cm wide, at a stocking density of 6 piglets per pen. All pens are located within the same house with concrete floor to ensure consistent conditions throughout the facility. Before the experiment, the pens were thoroughly cleaned and disinfected. The ventilation and lighting conditions in the pens were well-maintained. Daily cleaning and weekly spray disinfection were performed to keep the pens clean and tidy. The trial lasted for 32 days. During the trial, the piglets were provided with *ad libitum* access to both feed and water. Standard protocols for pig farm management and vaccination were adhered to in order to maintain the overall health and hygiene of the animals.

**Table 1:** Composition and nutrient levels of the basal diet (air-dry basis).

Basal ingredients	Content	Nutrient level	Content
Puffed soybean, %	5.00	Crude protein^c^, %	18.47
Soybean meal (46.0% CP), %	20.00	Crude fat^c^, %	5.75
Corn, %	46.30	Crude fiber^c^, %	2.27
Puffed corn, %	20.00	Neutral detergent fiber^c^, %	7.16
Soybean oil, %	2.00	Digestible neutral detergent fiber^b^, %	4.91
fish meal, %	3.00	Digestible carbohydrate^b^, %	12.35
Ca(H_2_PO_4_)_2_, %	0.90	Net energy^b^, Kcal/kg	2596.71
NaCl, %	0.42	Total digestible lysine^b^, %	1.21
Stone powder, %	0.40	Total digestible methionine+cystine^b^, %	0.63
Acidifier, %	0.40	Total digestible threonine^b^, %	0.59
L-lysine, % hydrochloride (98.5%), %	0.38	Total digestible tryptophan^b^, %	0.19
DL-methionine (99%), %	0.05	Total digestible valine^b^, %	0.79
Choline chloride (70%), %	0.05	Total digestible isoleucine^b^,%	0.69
Premix^a^, %	1.10	Calcium^c^, %	0.71
Total, %	100.00	Available phosphorus^b^, %	0.44

a The premix provided the following per kilogram of diet: VA, 3000 IU; VD, 1000 IU; VE, 100 IU; biotin, 0.20 mg; folic acid, 1.0 mg; Fe, 60 mg; Cu, 10 mg; Zn, 80 mg; Mn, 20 mg; Se, 0.10 mg.

b Calculated from the Chinese feed database, which provides tables of feed composition and nutritive values in China (2021 32nd edition).

c Analyzed nutrient composition.

### 2.3 Growth performance

Animals were weighed individually upon arrival and on day 32 and feed intake was recorded weekly throughout the study to calculate the average daily gain (ADG), the average daily feed intake (ADFI), and the feed-to-gain ratio (F/G). The feces and health of the piglets was visually checked daily and coded per pen on a scale ranging from 0 (extremely poor condition, long hair, diarrhea and high mortality) to 10 (normal feces, normal hair, excellent condition) (12). The incidence rate of diarrhea was calculated per group on a weekly basis by dividing the total number of pens with diarrhea by the total number of pens in each group, then multiplying by the duration of the trial in days, and finally multiplied by 100.

### 2.4 Sample collection

On day 32 of this experiment, after a 12 h fast, two piglets close to the average weight in each pen were chosen and blood was taken from the anterior vena cava. After standing at room temperature for 1 hour, serum was separated by centrifugation at 3,000 × g for 15 min and stored at −80°C for analysis of serum parameters. One of the two previously selected piglets (one from each pen) were then electrically stunned and euthanized, which complied with the Chinese Animal Welfare Guidelines and approved by the Animal Care and Use Committee of the Jiangxi Academy of Agricultural Sciences (2010-JXAAS-XM-01). An abdominal section was made and midsection of the duodenum, jejunum, and ileum was dissected, digesta of each segment were quantitatively collected and pH value was determined by pH meter (Testo 205, Testo instruments International Trading Ltd., Shanghai). Approximately 1 g of fresh digesta from ileum was taken, processed in a sterile cryotube, and stored at −80°C for bacteriological analysis.

### 2.5 Microscopic observation

The microstructures of SPEO, CSPEO and HSPEO were observed using a light microscope (Nikon Eclipse CI, Nikon Inc.). Image-Pro Plus 6.0 software (Media Cybernetics Inc., Rockville, MD, United States) was utilized to capture the optical images of these samples. Following drying in a carbon dioxide critical point dryer, the samples were affixed to the scanning electron microscope sample stage. Surface gold sputter coating was performed using the Eiko IB-5 Ion Coater (Eiko Co., Ltd., Japan). Subsequently, the samples were scanned under vacuum and an acceleration voltage of 15 kV using the JSM-6610LV scanning electron microscope (JEOL Ltd., Japan). Observation and image analysis were conducted using the DigitalMicrograph^®^ software (Gatan Inc., Pleasanton, CA, United States).

### 2.6 Serum parameters and intestinal endotoxins

Serum total protein (TP), albumin (ALB), globulin (GLB), urea nitrogen (BUN), total cholesterol (TC), triglyceride (TG), alanine aminotransferase (ALT), aspartate aminotransferase (AST), alkaline phosphatase (ALP) were measured with an automatic biochemical analyzer (Mindray BS-420, Shenzhen, Guangdong, China). Levels of serum immunoglobulin A (IgA), immunoglobulin G (IgG), immunoglobulin M (IgM), interleukin-4 (IL-4), interleukin-10 (IL-10), interleukin-1β (IL-1β), tumor necrosis factor α (TNF-α), interferon γ (IFN-γ), triiodothyronine (T3), and thyroxine (T4) were quantified using pig-specific ELISA kits obtained from Nanjing Jincheng Bioengineering Research Institute Co., Ltd. (Nanjing, China). The assay kit serial numbers were as follows: H108-1-2, H106-1-2, H109-1-2, H005-1-2, H009-1-2, H002-1-2, H052-1-2, H025-1-2, H222-1-2, and H223-1-2. Porcine insulin-like growth factor-1 (IGF-1) levels were measured using an ELISA kit from Shanghai Enzyme-linked Biotechnology Co., Ltd. (Shanghai, China), with assay kit serial number ml002344-2. All assays were conducted using a microplate absorbance reader (Multiskan SkyHigh, Thermo Fisher Scientific, Waltham, Massachusetts, United States) following the manufacturers’ protocols. Lipopolysaccharide (LPS) levels in the serum and chyme of the duodenum, jejunum, and ileum were detected using the limulus amoebocyte lysate method by Nanjing Jiancheng Bioengineering Institute Co., Ltd. (Nanjing, China).

### 2.7 Intestinal microflora

Total DNA from ileal contents was extracted using a kit (Thermo Fisher Scientific Inc. MA, United States), followed by quantification of DNA concentration using a micro UV–visible spectrophotometer-NanoDrop 2000 (Thermo Fisher Scientific Inc. MA, United States). The extracted DNA was subsequently stored at −20°C for further use. Specific PCR primers (Table 2) targeting bacterial 16S rRNA sequences were custom-designed using Primers software and synthesized by Shanghai Sangon Biotech Co., Ltd. (Shanghai, China). Real-time fluorescence quantitative PCR (Q-PCR) was conducted using custom synthetic primers to establish standard curves for target bacterial species. The expression levels of total bacteria, *Escherichia coli, Salmonella, Lactobacillus, and Bifidobacteria* were assessed following the protocol specified by the SYBR^®^ Green ProTaq HS pre-mixed qPCR kit (Hunan Aikerui Biotechnology Co., Ltd. Changsha, China). Cycle Threshold (Ct) values were utilized to determine the corresponding copy numbers based on the established standard curves. The results were reported as the quantity of 16S rRNA gene copies per gram of fresh weight.

**Table 2:** Information of primer sequences.

Target	Primer sequence (5′-3′)	Accession No.	Amplicon size (bp)
Total bacteria	F: ACTCCTACGGGAGGCAGCA	PP897811.1	174
	R: ATTACCGCGGCTGCTGG		
*Escherichia coli*	F: GCTTCCACTAACACACAC	CP151161.1	138
	R: ATAGGTTAATGAGGCGAAC		
*Bifidobacterium*	F: TCTTTGAGTTTTAGCCTTGCG	OQ552885.1	147
	R: GGGGAGCGAACAGGATTAG		
*Lactobacillus*	F: GAAACCAAGTGACCTACCCA	CP158455.1	103
	R: GCTACCCACAACTCATCCC		
*Salmonella*	F: TAACCTCACAACCCGAAGA	CP158241.1	103
	R: CAAGCTGAAAATTGAAACACA		

### 2.8 Statistical analysis

The data were analyzed using SPSS (IBM, SPSS Inc., Chicago, United States, IL, version 24.0). For this experiment, the dietary treatment replicate was treated as the experimental unit for growth performance, intestinal LPS content, pH value and ileum microbiota, and one piglet in each replicate was treated as the experimental unit for serum parameters. Statistical analysis was performed using the one-way ANOVA test. Significant differences between treatments were determined by Tukey’s multiple range test. The results were presented as group means and standard error of the mean (SEM). All the *p*-values were two-sided and the differences were considered statistically significant at *p <* 0.05, and statistically tendency at 0.05 < *p <* 0.10.

## 3 Results

### 3.1 The microstructural morphology of PEOs

**Figure 1:**
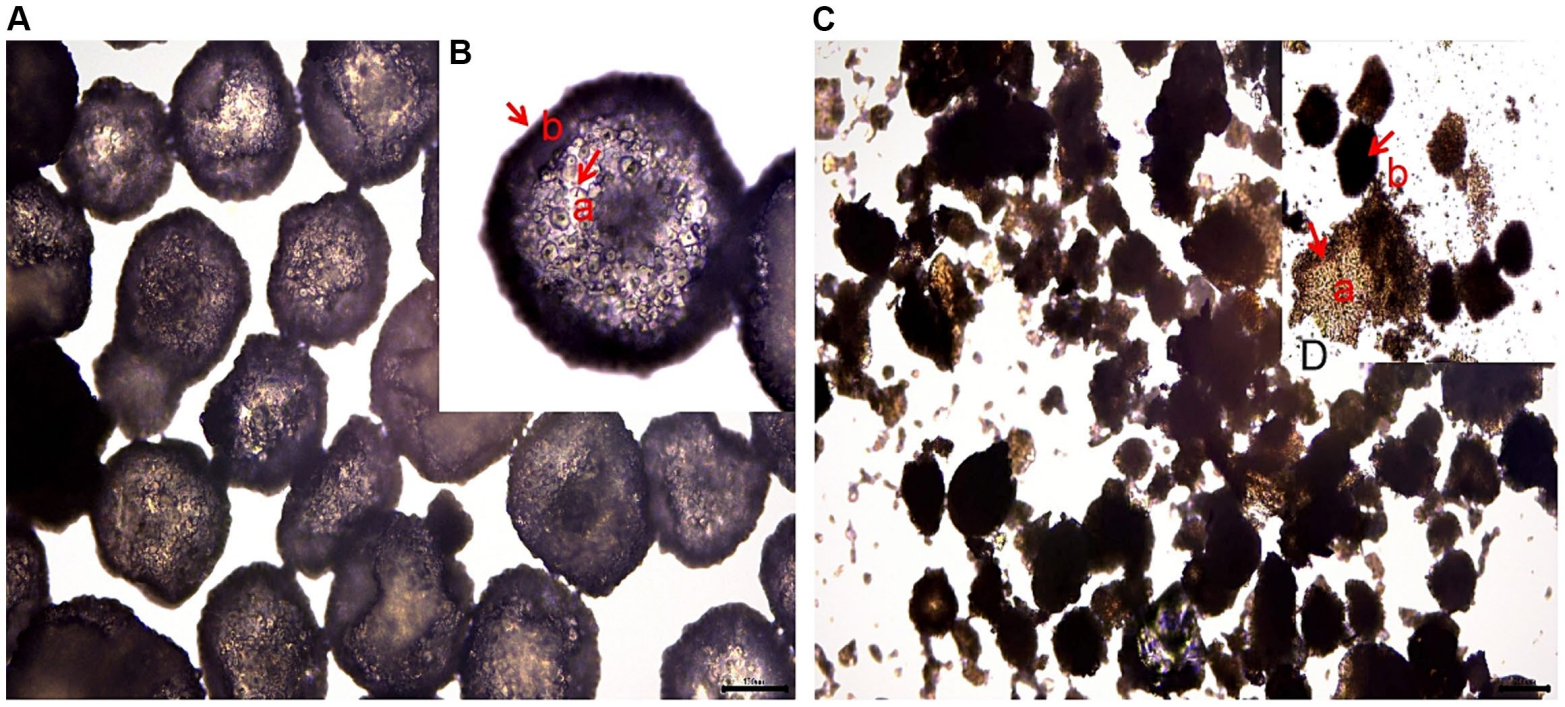
Morphology of PEO under an optical microscope. **(A)** The structure of HSPEO; **(B)** High-resolution close-up of the HSPEO structure; **(C)** The structure of SPEO; **(D)** High-resolution close-up of the SPEO structure. a, PEOs; b, wall matrix.

**Figure 2:**
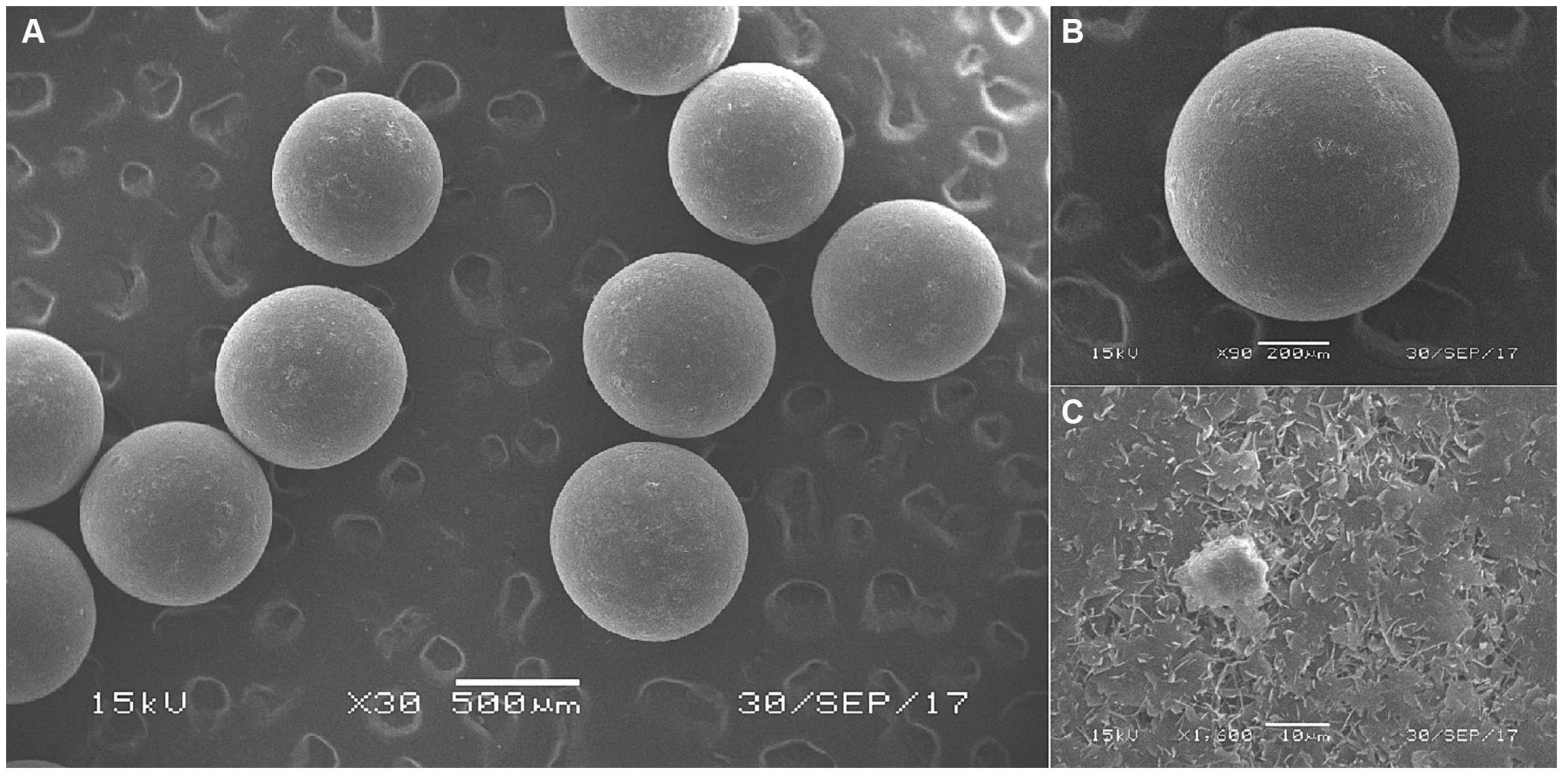
Surface morphology of CSPEO through scanning electron microscope. **(A–C)** The surface morphology of CSPEO at varying magnifications.

**Figure 3:**
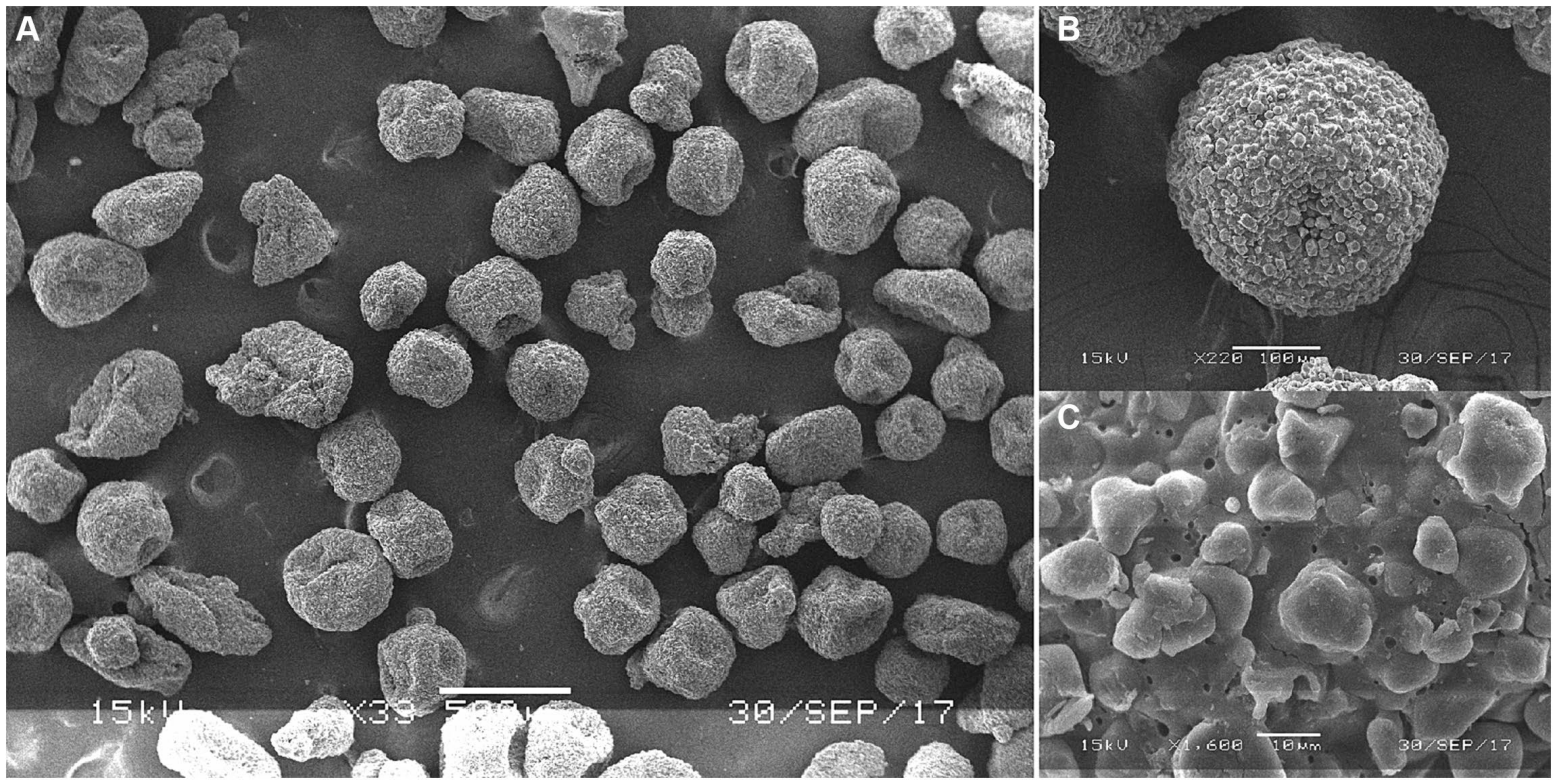
Surface morphology of HSPEO through scanning electron microscope. **(A–C)** The surface morphology of HSPEO at different magnifications.

Based on Figures 1–3, it becomes evident that the morphology of the SPEO particles is irregular, characterized by a heterogeneous distribution and an average particle size of approximately 500 μm. The edges of the particles appear indistinct, lacking well-defined boundaries. Moreover, noticeable tailing and agglomeration phenomena are observed, indicating a tendency for the particles to cluster together. CSPEO particles display a smooth and compact spherical shape, characterized by distinct boundaries and a flawless surface. They exhibit no evidence of fractures, depressions, or adhesion, while maintaining a plump and round appearance, with an average diameter of around 800 μm. However, the surface is too dense and opaque to be observed under a microscope. In contrast, HSPEO particles have a predominantly circular morphology, with an average particle size of approximately 300 μm. The PEO is enclosed by a thick and dense outer layer, which has a rough texture on its surface, characterized by irregularities and pores. Nevertheless, no visible indications of fractures, cracks, or depressions are observed. The boundaries remain well-defined, and there is no observed occurrence of particle trailing or coalescence, the particles retain their structural integrity.

SPEO microstructure showed a disorderly distribution of PEO with a significant portion located outside the wall matrix, similar to strawberry seeds (PEO) and pulp (wall matrix). In contrast, CSPEO demonstrates that while PEO is encapsulated within the wall matrix, it is relatively dispersed and embedded within the matrix, resembling the positioning of dragon fruit seeds (PEO) in the surrounding pulp (wall matrix). HSPEO particles closely resemble pure PEO compositionally, and no observable interaction between the wall matrix and the encapsulated PEO is present, akin to the relationship between pomegranate arils (PEO) and the peel (wall matrix).

### 3.2 Effect of PEOs on the growth performance

As shown in Table 3, the supplementation of antibiotics, SPEO, CSPEO, or HSPEO into the post-weaning diet of Tibetan piglets resulted in a significant reduction in diarrhea incidence and F/G ratio compared to the NC group (*p <* 0.05). There was a trend towards an increased final weight with HSPEO compared to the NC group (*p* = 0.054). Additionally, the ADFI in CSPEO and HSPEO group was significantly higher than that for SPEO (*p <* 0.05), while ADFI in both CSPEO and HSPEO did not show significant differences from the NC and PC groups (*p >* 0.05).

**Table 3:** Growth performance of weaned Tibetan piglets in different groups.

Items	NC	PC	SPEO	CSPEO	HSPEO	SEM	*p*-value
Initial weight, kg	5.14	5.10	5.13	5.18	5.16	0.020	0.839
Final weight, kg	11.62	12.33	11.86	12.15	12.38	0.100	0.054
ADFI, g/d	435.71^ab^	437.24^ab^	417.60^b^	432.30^ab^	448.58^a^	3.389	0.046
ADG, g/d	203.75^b^	225.82^a^	210.09^ab^	217.84^ab^	225.50^a^	2.778	0.023
F/G	2.14^a^	1.94^b^	1.99^b^	1.99^b^	1.99^b^	0.022	0.020
Diarrhea rate, %	6.38^a^	1.30^c^	4.30^b^	4.56^b^	4.04^b^	0.387	<0.001

Values are represented as the mean and SEM (*n* = 4). ^a–b^ Means within a row without a common superscript differ significantly (*p* < 0.05). SEM, standard error of the mean; NC, negative control; PC, positive control; SPEO, solidified plant essential oils; CSPEO, cold spray-coated plant essential oils; HSPEO, hot spray-coated plant essential oils; ADFI, average daily feed intake; ADG, average daily gain; F/G, feed to gain ratio.

### 3.3 Effect of PEOs on the serum parameters

Table 4 showed the effects of SPEO, CSPEO, and HSPEO on serum biochemical indices. There were no significant differences observed in serum TP, ALB, GLB, BUN, TC, TG, AST, ALT, ALP levels among the different treatment groups (*p >* 0.05). As presented in Table 5, compared with the NC group, the supplementation of SPEO, CSPEO and HSPEO had a significant increase in serum IgG content (*p <* 0.05). Compared with the NC and PC groups, SPEO, CSPEO and HSPEO exhibited a significant reduction in serum LPS and IFN-γ levels (*p <* 0.05). Supplementation with both HSPEO and SPEO significantly increased serum IGF-1 concentration in weaned piglets compared with the NC group (*p <* 0.05). Serum TNFα concentration in SPEO, CSPEO and HSPEO groups showed a declining trend compared with NC group (*p* = 0.096). Notably, the serum IgM and IL-10 levels in the HSPEO group were significantly higher than those in both the NC and PC groups (*p <* 0.05). There were lower serum IL-1β concentration and higher serum IL-4 concentration in HSPEO group than those in the NC and PC groups (*p <* 0.05). However, no significant differences were observed in serum IgA, T3, and T4 levels among the different groups (*p >* 0.05).

**Table 4:** Serum metabolic indexes of weaned Tibetan piglets in different groups.

Items	NC	PC	SPEO	CSPEO	HSPEO	SEM	*p*-value
TP, g/L	40.66	42.83	47.77	47.14	50.21	2.399	0.750
ALB, g/L	17.06	18.45	19.76	18.93	20.36	0.859	0.810
GLB, g/L	23.60	24.38	28.01	28.20	29.85	1.647	0.742
BUN, mmol/L	3.13	3.07	3.51	3.30	2.85	0.110	0.468
TC, mmol/L	1.72	1.67	1.74	1.76	1.39	0.100	0.752
TG, mmol/L	0.43	0.38	0.40	0.49	0.45	0.019	0.335
AST, U/L	44.47	36.67	42.09	48.07	35.82	3.294	0.724
ALT, U/L	33.01	34.92	32.48	35.82	28.61	2.080	0.831
ALP, U/L	148.52	179.22	164.30	200.00	179.16	9.602	0.489

Values are represented as the mean and SEM (*n* = 8). SEM, standard error of the mean; NC, negative control; PC, positive control; SPEO, solidified plant essential oils particles; CSPEO, cold spray-coated plant essential oils; HSPEO, hot spray-coated plant essential oils; TP, total protein; ALB, albumin; GLB, globulin; BUN, blood urea nitrogen; TC, total cholesterol; TG, total triglycerides; AST, aspartate aminotransferase; ALT, alanine transaminase; ALP, alkaline phosphatase.

**Table 5:** Serum immune and hormonal indexes of weaned Tibetan piglets in different groups.

Items	NC	PC	SPEO	CSPEO	HSPEO	SEM	*p*-value
IgA, g/L	1.11	1.14	1.20	1.18	1.18	0.012	0.127
IgG, g/L	18.15^b^	19.73^b^	21.75^a^	22.56^a^	23.67^a^	0.386	<0.001
IgM, (g/L)	2.09^b^	2.13^b^	2.24^ab^	2.27^ab^	2.38^a^	0.029	0.010
LPS, EU/mL	0.57^a^	0.54^a^	0.46^b^	0.47^b^	0.42^b^	0.010	<0.001
IFN-γ, pg/mL	49.64^a^	47.70^a^	42.07^b^	41.76^b^	37.50^b^	0.999	<0.001
IL-1β, pg/mL	33.91^a^	34.18^a^	31.73^ab^	29.83^ab^	27.49^b^	0.819	0.047
TNFα, pg/mL	65.21	63.98	60.11	59.30	54.16	1.388	0.096
IL-4, pg/mL	7.43^b^	7.12^b^	8.46^ab^	8.71^ab^	9.99^a^	0.271	0.007
IL-10, pg/mL	13.04^c^	14.22^bc^	16.96^ab^	17.69^a^	19.47^a^	0.549	<0.001
IGF-1, ng/mL	169.49^b^	192.78^ab^	215.99^a^	195.36^ab^	219.85^a^	4.662	0.003
T3, (ng/mL)	0.52	0.53	0.54	0.53	0.56	0.004	0.172
T4, ng/mL	51.58	53.26	53.95	53.08	54.09	0.354	0.176

Values are represented as the mean and SEM (*n* = 8). ^a–c^ Means within a row lacking a common superscript differ significantly (*p* < 0.05). SEM, standard error of the mean; NC, negative control; PC, positive control; SPEO, solidified plant essential oils particles; CSPEO, cold spray-coated plant essential oils; HSPEO, hot spray-coated plant essential oils; IgA, immunoglobulin A; IgG, immunoglobulin G; IgM, immunoglobulin M; LPS, lipopolysaccharide; IFN-γ, interferon γ; IL-1β, interleukin-1β; TNFα, tumor necrosis factorα; IL-4, interleukin 4; IL-10, interleukin10; IGF-1, insulin-like growth factor1; T3, total triiodothyronine; T4, total tetraiodothyronine.

### 3.4 Effect of PEOs on the intestine LPS content and pH value

As shown in Table 6, compared with the NC group, SPEO, CSPEO, and HSPEO significantly reduced the content of LPS in the duodenum (*p <* 0.05). Additionally, the LPS levels in the SPEO and HSPEO groups were significantly lower than those in the PC group (*p <* 0.05). Compared with the NC group, SPEO, CSPEO, and HSPEO exhibited a declining tendency in jejunum LPS content (*p* = 0.087). The jejunum pH value in the CSPEO and HSPEO groups was significantly lower than that in the PC and NC groups (*p <* 0.05). Furthermore, the addition of HSPEO in the diet led to a significant reduction in duodenum pH value (*p <* 0.05) and showed a declining tendency in ileum pH value (*p* = 0.076). However, there were no significant differences in the ileum LPS content among the different groups (*p >* 0.05).

**Table 6:** Intestinal LPS and pH value of weaned Tibetan piglets in different groups.

Items	NC	PC	SPEO	CSPEO	HSPEO	SEM	*p*-value
Duodenum							
LPS, EU/mg.pro	0.36^a^	0.34^ab^	0.29^cd^	0.31^bc^	0.26^d^	0.009	<0.001
pH value	6.02^a^	6.00^a^	5.82^a^	5.90^a^	5.58^b^	0.042	0.004
Jejunum							
LPS, EU/mg.pro	0.34	0.32	0.31	0.31	0.30	0.005	0.087
pH value	6.37^a^	6.48^a^	6.35^a^	5.99^b^	5.88^b^	0.045	<0.001
Ileum							
LPS, EU/mg.pro	0.28	0.26	0.23	0.24	0.23	0.009	0.295
pH value	6.82	7.04	6.96	6.86	6.58	0.055	0.076

Values are represented as the mean and SEM (*n* = 4). ^a–d^ Means within a row lacking a common superscript differ significantly (*p* < 0.05). SEM, standard error of the mean; NC, negative control; PC, positive control; SPEO, solidified plant essential oils particles; CSPEO, cold spray-coated plant essential oils; HSPEO, hot spray-coated plant essential oils; LPS, lipopolysaccharide.

### 3.5 Effect of PEOs on the ileum microbiota

As shown in Table 7, it was observed that the abundance of *Bifidobacterium* in the ileum was higher in the SPEO, CSPEO, and HSPEO groups than those in the NC group (*p <* 0.05). Supplementation HSPEO significantly increased the abundance of *Lactobacillus* (*p <* 0.05) and showed a noticeable trend of increased total bacteria abundance (*p* = 0.058) compared to the NC and PC groups. No differences were detected in the abundance of *E. coli* and *Salmonella* among the groups (*p >* 0.05).

**Table 7:** Ileum microbiota of weaned Tibetan piglets in different groups lg (copies/g).

Items	NC	PC	SPEO	CSPEO	HSPEO	SEM	*p*-value
Total bacteria	11.49	11.30	11.39	11.30	11.64	0.324	0.058
*Lactobacillus*	9.67^b^	9.34^b^	9.28^b^	9.46^b^	10.32^a^	0.045	0.002
*Bifidobacterium*	9.62^b^	9.88^ab^	10.14^a^	10.30^a^	10.30^a^	0.111	0.019
*Escherichia coli*	8.31	6.80	7.48	7.20	7.12	0.076	0.396
*Salmonella*	6.90	6.17	6.72	6.59	6.00	0.248	0.309

Values are represented as the mean and SEM (*n* = 4). ^a–b^ Means within a row lacking a common superscript differ significantly (*p* < 0.05). SEM, standard error of the mean; NC, negative control; PC, positive control; SPEO, solidified plant essential oils particles; CSPEO, cold spray-coated plant essential oils; HSPEO, hot spray-coated plant essential oils.

## 4 Discussion

PEOs are characterized by their highly volatile and sensitive to both light and temperature, which can diminish their efficacy when orally administered (19). Upon oral administration, these oils are rapidly absorbed in the gastrointestinal tract, thereby limiting their interaction with intestinal microflora and diminishing therapeutic outcomes (20, 21). Microencapsulation technology has emerged as a sophisticated strategy to address these challenges (22, 23). By encapsulating PEOs within protective matrices, this technology mitigates volatilization of active components, enhances stability against environmental stressors such as light and heat, ensures product integrity during processing and storage, facilitates targeted release in the intestine, and prolongs their residence time for enhanced interaction with intestinal microflora (24, 25).

The microstructural morphology of microcapsules plays a crucial role in determining the encapsulation status and microencapsulation effects of PEOs. Scanning electron microscopy analysis in this study revealed that HSPEO exhibited a complete encapsulation structure, with each individual microcapsule enveloped by a hydrophilic wall matrix. This structure ensures comprehensive coverage of the hydrophobic essential oil as the core material, providing heat resistance and stability to prevent loss and degradation of the core material. The presence of small pores on the membrane allows for the passage of water molecules and enzymes, facilitating the simultaneous dissolution of the inner and outer membranes and enabling the complete release of the essential oil. In contrast, CSPEO appeared as smooth and compact particles with a dense structure, limiting effective encapsulation of the oil molecules and hindering their full and efficient release. The disordered distribution of essential oil within the CSPEO microstructure hampers its efficient encapsulation within the wall matrix. The HSPEO microparticles were slightly smaller and had a narrower size distribution compared with SPEO and CSPEO. This size allows the microparticles to be blended with the feed easily and thus provides convenience for industrial applications (25).

Thyme phenol and cinnamaldehyde possess unique aromas that may impact the feeding behavior of animals, particularly during the weaning period when piglets are highly sensitive to the physical attributes, odor, and taste of their feed. This study discovered that the addition of PEO significantly increased the final body weight of piglets while reducing the F/G, consistent with the findings of Zou’s research (26). These results suggest that PEOs may have a positive influence on improving feed utilization in piglets. Previous research indicated that PEOs can affect the expression of neuropeptide mRNA, resulting in heightened appetite and improved feeding capacity in animals (27). However, our study revealed that it was noteworthy that ADFI was notably lower in the SPEO group relative to the HSPEO group, this implied that HSPEO can effectively enhance piglet feed intake, whereas the insufficient masking effect of SPEO on taste might hinder its appetite-stimulating properties. PEO has been found to alleviate digestive disorders and gastritis, exhibiting anti-ulcer effects (28). Consequently, they can help mitigate intestinal stress during the weaning period and enhance piglet growth performance. Additionally, PEO can regulate piglet endocrine functions by promoting the secretion of IGF-1, T3, and T4 hormones (29, 30). IGF-I, as a synthetic metabolic hormone, facilitates the absorption of amino acids and glucose by cells, fosters protein, fat, and glycogen synthesis, inhibits protein degradation, and promotes piglet growth (31). Serum IGF-I levels have been positively correlated with pig weight and weight gain (32). The results of this study demonstrate that PEO increased the serum IGF-1 content in piglets, while exhibiting no significant impact on T4 and T3 levels.

After weaning, the transition from maternal milk to solid feed in piglets, combined with inadequate gastric acid secretion, can result in an elevated pH value in the gastrointestinal tract. This high pH value in the intestines can negatively impact the activity of carbohydrate-digesting enzymes, leading to incomplete carbohydrate digestion and providing a favorable environment for harmful bacteria, ultimately causing piglet diarrhea. Research conducted by Mei et al. (33) has demonstrated a significant positive correlation between *E. coli.* Quantity and pH value. Our results revealed that HSPEO effectively reduced the pH values in these sections of the gastrointestinal tract. Lower pH value was found to decrease the adhesion of pathogenic microorganisms and inhibit the virulence factors of harmful bacteria. Concurrently, enzyme activity increased under low pH value, resulting in improved utilization of carbohydrates and a reduction in growth substrates for harmful bacteria (34). Moreover, this study found that HSPEO significantly increased the abundance of Bifidobacterium and Lactobacillus in the ileum of piglets, which aligns with the findings reported by Li et al. (35). Bifidobacterium and Lactobacillus are capable of producing short-chain fatty acids such as lactic acid, acetic acid, propionic acid, butyric acid, and antimicrobial factors. These beneficial bacteria competitively inhibit the proliferation of pathogenic bacteria and play a crucial role in regulating the immune response and maintaining intestinal health (36, 37). The significant decrease in pH value observed in the duodenum and jejunum, as well as the decreasing trend in the ileum observed in our study, can be attributed to the promotion of beneficial bacteria by PEOs and the notable increase in propionic acid concentration in the caecum (38). Consequently, the impact of PEOs on piglet growth performance appears to be multifaceted. The microencapsulation of thymol and cinnamaldehyde helps reduce odor and enhance feed intake, while also stimulating the secretion of the IGF-I hormone. Moreover, the reduction in intestinal pH value and the increase in the abundance of beneficial bacteria synergistically improve feed utilization and promote growth in piglets.

Thyme phenol and other PEOs have been found to effectively inhibit the growth and reproduction of various harmful bacteria, leading to a reduction in the secretion of intestinal toxins by these pathogens (39). Cinnamaldehyde has also been shown to suppress harmful microorganisms in the digestive tract and promote the proliferation of beneficial microorganisms (40). This promotes an increase in butyric acid content in the small intestine, which helps protect and repair the intestinal mucosal epithelium, preventing and controlling diarrhea in piglets (6).

Through various combinations and encapsulation techniques, it is possible to reduce the required amount of plant essential oils (PEOs) while still achieving the desired benefits. Our experimental results demonstrated that different encapsulated forms of PEOs significantly reduced the incidence of diarrhea in weaned piglets, consistent with the findings of Li et al. (40). This reduction may be attributed to the increased relative abundance of Lactobacillus (41). The PEO dose used in our study to reduce diarrhea in piglets was significantly lower than that used by Shen (42), who administered 600 mg/kg of cinnamaldehyde. This highlights that effective encapsulation and the combination of active PEO components are crucial for maximizing the benefits. However, our study’s dose was higher than that used by Li et al. (35), who found that adding 150 mg/kg of encapsulated thyme phenol and cinnamaldehyde complex PEOs to post-weaning pig feed reduced the incidence of diarrhea and improved piglet growth performance. This discrepancy may be due to differences in the composition of essential oils and the breeds of animals used.

The results of this study showed that the addition of PEO did not significantly alter the levels of TP, ALB, GLB, Urea, TC, and TG in piglet serum. This suggests that PEO does not notably influence protein and lipid metabolism in piglets, consistent with the findings of Shen (42). While some research, such as Raskovic et al. (43), has indicated that high doses of PEO may affect liver function in animals, our study found no significant changes in AST, ALT, and ALP levels in piglets. This implies that the PEO dosage used in our study does not harm liver function. Therefore, the concentration of PEO administered was deemed suitable and did not cause adverse effects on the liver. These findings confirm that the PEO dosage used is both safe and non-disruptive to protein and lipid metabolism as well as liver function in piglets.

The addition of PEOs to the diet has been shown to enhance the body’s immune response (24). Similarly, the supplementation of a thymol and cinnamaldehyde complex in the post-weaning piglet diet has been found to increase the levels of natural antibodies such as IgA, IgG, and complement factors C3 and C4 in the serum (44). Consistent with these findings, our study revealed that supplementing the piglet diet with 300 mg/kg HSPEO significantly elevated the levels of IgA, IgG, and IgM in piglet serum. This effect is likely due to the ability of PEOs to activate the immune response, thereby increasing the peripheral blood lymphocyte transformation rate (45). Immune cell proliferation and activation play critical roles in the development and progression of inflammation. The constituents of PEOs have been found to possess anti-inflammatory properties, including the inhibition of immune cell proliferation and the regulation of cytokine expression (46). This leads to the promotion of the release of the anti-inflammatory factor IL-10 from macrophages and the inhibition of pro-inflammatory factors such as TNF-α, IL-1β, and IL-6, ultimately alleviating the inflammatory response (47).

Our study indicates that different encapsulation forms of PEO can reduce the release of pro-inflammatory factors (IFN-γ and IL-1ß). However, only HSPEO, which we hypothesize can achieve high concentrations within the body, can enhance the release of anti-inflammatory factors (IL-4 and IL-10). Early weaning has been observed to elevate cortisol levels in serum and increase the expression of cortisol release receptors in the intestinal mucosa (48). This triggers an inflammatory response, leading to an upregulation of mRNA expression for pro-inflammatory factors such as TNF-ɑ, IL-1ß, and IL-6 in the intestinal mucosa. Excessive production of pro-inflammatory cytokines can disrupt the structure and function of the intestinal epithelium, causing a decrease in paracellular permeability of intestinal cells due to increased expression of IFN-γ and TNF-α. Consequently, toxin absorption into the bloodstream intensifies (49). The addition of thymol and other complex PEOs to the diet has been found to significantly reduce mRNA expression of TNF-ɑ in the intestine (50). It also diminishes the expression of IL-1ß and IL-6 mRNA, lowers the content of IL-1 and IL-6 in the serum, and mitigates colon tissue damage (51). LPS is an essential component of the cell wall of Gram-negative bacteria, and its levels in the bloodstream can reflect the integrity of the intestinal mucosal barrier (52). Our study has shown that the supplementation of PEOs is linked to significant reductions in both serum and duodenal concentrations of LPS. This reduction can be attributed to two key factors we have identified. Firstly, PEO has been shown to effectively balance the gut microbiota, which reduces the release of their toxins into the gut environment. Studies have indicated a negative correlation between LPS concentration and the presence of *Lactobacillus* (53). Secondly, our results suggest that PEO is able to reduce the secretion of inflammatory factors, thereby improving the integrity of the intestinal mucosal barrier. This improvement reduces the absorption of LPS into the bloodstream. These findings are significant given the well-established link between elevated LPS levels and a range of negative health outcomes, including inflammation, metabolic disorders, and other diseases (54). However, it is noteworthy that PEO supplementation did not significantly affect LPS levels in the jejunum and ileum. One possible explanation for this observation is that the jejunum and ileum have different microbiota compositions and immune environments compared to the duodenum. The differential impact of PEO on these sections of the intestine may be due to variations in local bacterial populations or differences in the intestinal permeability and immune response in these regions.

## 5 Conclusion

Microencapsulation of PEOs effectively solidifies and stabilizes these plant-derived oils. The microstructural morphology showing that HSPEO successfully encapsulates the essential oils within the wall matrix. The inclusion of 300 mg/kg of SPEO, CSPEO, and particularly HSPEO in the post-weaning diet of Tibetan piglets significantly reduces diarrhea rates, improves growth, enhances immune function, and optimizes gut microbiota composition. This makes HSPEO a promising dietary supplement for swine production. However, this study did not investigate the effects of HSPEO on the intestinal mucosal barrier, including the physical, chemical, and immune barriers. Future research should explore these aspects to comprehensively evaluate the role of HSPEO in gut health.

## Data Availability

The datasets generated for this study are available on request to the corresponding author.
